# Mechanism of Phellodendron and Anemarrhena Drug Pair on the Treatment of Liver Cancer Based on Network Pharmacology and Bioinformatics

**DOI:** 10.3389/fonc.2022.838152

**Published:** 2022-04-07

**Authors:** Xiaofeng Ruan, Wenyuan Li, Peng Du, Yao Wang

**Affiliations:** ^1^ College of Traditional Chinese Medicine, Hubei University of Traditional Chinese Medicine, Wuhan, China; ^2^ Department of Rehabilitation Medicine, Xiangyang Central Hospital, Affiliated Hospital of Hubei University of Arts and Science, Xiangyang, China; ^3^ Department of Anesthesiology, Renmin Hospital of Wuhan University, Wuhan, China; ^4^ Department of Infectious Diseases, Renmin Hospital of Wuhan University, Wuhan, China

**Keywords:** Phellodendron and Anemarrhena drug pair, liver cancer, network pharmacology, bioinformatics, molecular docking

## Abstract

**Background:**

This study aims to explore the key targets and signaling pathways of the traditional Chinese medicine Phellodendron and Anemarrhena drug pair (PADP) for the treatment of liver cancer.

**Methods:**

Firstly, bioinformatics technology was used to analyze GSE62232 gene chip to obtain the differential genes of liver cancer. A network pharmacology technology was used to find the active components of PADP and their targets. Secondly, the differential genes were imported into STRING database to draw a PPI network, and network topology structure map combined with Cytoscape software. And the R language was used to identify differential gene targets and pathways through GO and KEGG pathway enrichment analysis. In addition, AutoDock Vina was used for molecular docking of core targets and core compounds. Moreover, GEPIA online analysis tool was used to perform survival analysis of the core target genes. Finally, RT-PCR was used to verify the changes of key target genes. CCK−8 assay was performed to detect cell proliferation. Flow cytometry was performed to detect the cell cycle and apoptotic. Transwell invasion assay was performed to detect cell invasion.

**Results:**

Firstly, a total of 21,654 genes were obtained. After screening, 1019 differential genes were obtained, including 614 down-regulated genes and 405 up-regulated genes. Furthermore, after screening by ADME standards, 52 active ingredients were obtained, of which 37 were Phellodendron and 15 were Anemarrhena. And a total of 36 differential genes have been identified, including 13 up-regulated genes and 23 down-regulated genes. Moreover, through enrichment analysis, we found that PADP may treat liver cancer through multiple channels and multiple pathways including the p53 signaling pathway, IL-17 signaling pathway, TNF signaling pathway, Toll-like receptor signaling pathway and so on. Secondly, the molecular docking results showed that there was certain affinity between the core compounds and core target genes. In addition, GEPIA online analysis showed that ESR1, AR, CCNB1, CDK1, AKR1C3 and CCNA2 might become potential target genes for the survival and prognosis of PADP for the treatment of liver cancer. Finally, it was found that PADP could up regulate genes ESR1 and AR, down regulate genes CCNB1, CDK1, AKR1C3, and CCNA2. PADP could promote the apoptosis of liver cancer cells, shorten the cell cycle, and inhibit the proliferation and invasion of liver cancer cells.

**Conclusion:**

PADP may treat liver cancer through multiple targets, multiple channels, and multiple pathways, thereby suppressing cancer cells and improving the living quality of patients.

## Introduction

As we all know, liver cancer is the third leading cause of cancer-related deaths, especially in developing countries, where the incidence has increased in recent years. Globally, there are approximately 630,000 new liver cancer cases each year, more than half of which occur in China (1). With high morbidity and death rates, liver cancer has become one of the most common cancers in the world (2). At present, the prognosis of liver cancer after chemotherapy, radiotherapy, and surgery is the main focus of medical research (3). Traditional Chinese medicine plays a vital role in the treatment of early liver cancer, reducing the complications of radiotherapy, chemotherapy and postoperative surgery in the advanced liver cancer, and improving the clinical symptoms of patients. The combination of Phellodendron and Anemarrhena is a well-known drug pair of traditional Chinese medicine, which has significant effect in the treatment of liver cancer with damp-heat and liver-kidney yin deficiency. Phellodendron has the effects of clearing away heat and dampness, purging fire and detoxifying, nourishing yin and reducing fire. Modern pharmacological studies have shown that Phellodendron contains many active ingredients and exerts a wide range of physiological activities. It includes anti-tumor activity, antibacterial and immunosuppressive (4). At the same time, as a traditional Chinese herbal medicine, Anemarrhena possesses the effects of nourishing yin, moistening dryness, clearing heat and purging fire (5). Studies have shown that Anemarrhena has protective effect on the liver (6) and has anti-tumor effects (7). Overall, PADP has positive effect on inhibiting the growth of cancer cells in patients with liver cancer, alleviating clinical symptoms of patients, improving their living quality, and increasing the survival period of patients. Due to the complex composition of traditional Chinese medicines (8), it is difficult for traditional pharmacological research methods to systematically explain the mechanism of action and signaling pathways of PADP on the treatment of liver cancer. Therefore, the mechanism of PADP on the treatment of liver cancer has not yet been clarified.

Traditional Chinese medicine network pharmacology is a technology that integrates multiple disciplines such as systems biology, pharmacology, and computer analysis (9–11). It connects drugs and diseases from an overall perspective, and provides new strategies for exploring the mechanism of traditional Chinese medicine and developing new drugs. At present, the research and development of traditional Chinese medicine urgently necessitates the application of network pharmacology of traditional Chinese medicine, which coincides with the thinking model of the overall view of traditional Chinese medicine. Bioinformatics is a new discipline based on the intersection of molecular biology and mathematics, computer science, statistics and other disciplines. It can be used to study the relationship and laws of biological genes and diseases. In addition, it has rapidly developed into the most attractive frontier of life sciences today (12, 13). Molecular docking plays an important guiding role in the development and design of drugs and may provide keen insights in protein function prediction and other important issues.

This article used network pharmacology combined with bioinformatics technology to explore the mechanism and signaling pathways of PADP for the treatment of liver cancer and used molecular docking technology and cell assays to verify the results.

## Materials and Methods

### Data Acquisition and Annotation of GEO Gene Chip

The liver cancer related targets were obtained from the GEO database (https://www.ncbi.nlm.nih.gov/geo/) developed by the National Center for Biotechnology Information, and the original gene chip data number GSE62232 was downloaded. The gene chip data set, which has a total of 91 samples, including 81 liver tumor samples from liver cancer patients and 10 normal human liver tissue samples, was derived from the gene chip sequencing of the University of Battieril, France. And the samples were detected using the Affymetrix U133plus v2 array (GPL570) platform. Finally, the Perl language was used to annotate the GEO data, that was, converting the probe matrix into a gene matrix.

### Acquisition of Differential Genes

Firstly, the above genes were divided into tumor group and normal group by Perl language. Then the limma software package in R language was used to analyze the gene chip data to obtain the gene differential expressions (DEGs) between tumor group and normal group in GPL570. The logFC and adjusted *P* value were used as the screening conditions. Genes that met |LogFC| ≥1 and adjusted *P*<0.05 were considered as significantly differentially expressed genes. The volcano plot and heatmap plot of the corresponding differential genes were obtained using the pheatmap package in R language.

### Acquisition of Chemical Components and Targets of PADP

In this study, the Traditional Chinese Medicine Systems Pharmacology Database and Analysis Platform (TCMSP) (14) (http://tcmspw.com/tcmsp.php) was used to obtain the active chemical components and to predict targets of Phellodendron and Anemarrhena. In the reference standard for evaluating whether a compound can become a drug, oral bioavailability (OB) and drug-like (DL) are two commonly used indicators (15). Here, we used OB ≥ 30% and DL ≥ 0.18 as the screening threshold to obtain the active components of Phellodendron and Anemarrhena. At the same time, Perl language was used to predict the target genes corresponding to the above active components in the TCMSP database. Combined with the UniProt database (16) (http://www.uniprot.org/, update in 2018-04-10), the full name of the retrieved gene was converted into its official name (gene symbol).

### Compounds-Targets Network Diagram

The active compounds selected by the above TCMSP database and their predicted target genes were imported into Cytoscape_v3.6.1 software (17) (http://www.Cytoscape.org/) to construct a compounds-targets network diagram.

### Intersection of Disease Genes and Drug Genes

The target genes predicted by the effective components of Phellodendron and Anemarrhena were intersected with the up-regulated and down-regulated genes respectively in the differential genes of liver cancer. Using the “VennDiagram” package in R to draw a Venn diagram.

### Construction of Traditional Chinese Medicine Compound Regulation Network

The target genes predicted by the active ingredients of PADP and the differential genes of liver cancer were intersected and imported into the Cytoscape_v3.6.1 software to construct an ingredients–targets network diagram of PADP in the treatment of liver cancer. Then, the above-mentioned intersection genes were imported into the STRING database (https://string-db.org/) for protein-protein interaction (PPI) analysis, the txt file was downloaded and copied to excel for annotation, and then imported into Cytoscape software to draw the core genes PPI network diagram. At the same time, the intersection genes were introduced into R language to draw core genes bar graph.

### PPI and Network Topology Analysis

The above intersection genes were input into Cytoscape software, and the “Bisogenet” package in the software was used for PPI analysis to obtain a PPI network diagram, while the CytoNCA package in the software was used for network topology analysis. And the DTP, BIOGRID, HPRD, INTACT, MIMT, BIND were selected as data sources.

### GO Analysis and KEGG Pathway Enrichment Analysis

Combining Gene Ontology (GO) (http://GeneOntology.org/) and Kyoto Encyclopedia of Genes and Genomes (KEGG) (https://www.kegg.jp/kegg/), “colorspace”, “stringi”, “ggplot2” packages in R language and “DOSE”, “clusterProfiler”, “enrichplot” packages in BiocManager database (18) (http://www.bioconductor.org/) were applied. Selected the filter condition organization = “hsa” and set “P vulue Cutoff = 0.05 and Q vulue Cutoff = 0.05”. Cellular component (CC), biological process (BP) and molecular function (MF) biological processes of GO enrichment analysis were shown in the form of bubble charts, and KEGG pathway enrichment analysis was shown in the form of bar charts.

### Construction of KEGG Relationship Network

The pathway ID number and the genes enriched by the pathway were respectively imported into Cytoscape software. And then the number of adjacent nodes in the network was calculated and the size of the nodes in the network was determined according to the number of adjacent nodes to construct a KEGG relationship network.

### Molecular Docking Verification of Core Compounds and Core Target Genes

Firstly, select the top 10 core compounds and download the two-dimensional structure diagrams of the compounds from the PubChem database, import Chem3D software to draw the three-dimensional structure diagrams of the core compounds and optimize the energy, save them in mol2 format, and then import the AutoDockTools-1.5.6 software to add charge, display rotatable keys, and save as pdbqt format. Secondly, download the protein crystals corresponding to the top 10 down-regulated genes and the top 5 up-regulated genes in the PDB database, import Pymol software to remove water molecules and heteromolecules, and then import AutoDockTools-1.5.6 software to add hydrogen atoms and charge operations, save to pdbqt format, and then import Discovery Studio 3.5 Client software to search for active pockets. Finally, the above core compounds were used as ligands, and core target gene corresponding proteins were used as receptors for molecular docking, and the results of interaction force were analyzed and interpreted using Discovery Studio 3.5 Client.

### Survival Analyses for Hub Genes

The association between overall survival (OS) and the hub genes was determined using the online tool GEPIA. The lower 25% and upper 75% of gene expression were set as the standard for analysis. In the present study, HCC patients were categorized into 2 groups based on the median expression values of hub genes. We calculate the hazards ratio based on Cox PH Model and add the 95% CI as dotted line. Log-rank test results with *P*<0.05 were regarded as statistically significant.

### Quantitative Real-Time PCR (RT-PCR) to Detect mRNA Expression

RT-PCR was used to verify the effect of PADP on the changes of key target genes in human hepatocarcinoma HepG2 cell line and Huh7 cell line, include the down-regulated genes ESR1 and AR, and up-regulated genes CCNB1, CDK1, AKR1C3, and CCNA2. HepG2 and Huh7 cells were cultured in Dulbecco’s Modified Eagle Medium (DMEM, Gibco, USA) mixed with 10% fatal bovine serum (FBS, Gibco, USA). PADP was dissolved in PBS and then filtered with a 0.44um filter. HepG2 and Huh7 were then treated with PADP solution (200mg/L, half Phellodendron and half Anemarrhena) for 24h. The PCR procedure followed the previously published steps (19, 20). Total RNAs from HepG2 cell line were isolated by using RNAiso Plus (TaKaRa, Dalian, China) according to the manufacturer’s protocol. The cDNAs were produced with a Prime-Script RT reagent kit and incubated at 37°C for 15 min and at 85°C for 5 s. Quantitative real-time PCRs were performed using a StepOnePlus device (Applied Biosystems) at 95°C for 10 s, followed by 40 cycles at 95°C for 5 s and at 60°C for 20 s, according to the instructions for the SYBR Premix Ex Taq kit (TaKaRa, Dalian, China). The data were analyzed by the ^2−ΔΔCT^ method. All the primer sequences (**Table 1**) were designed and synthesized by Tsingke (Wuhan, China). GAPDH was set as the housekeeping gene.

**Table 1:** Primers for RT-PCR.

Genes	Forward (5′-3′)	Reverse (5′-3′)
**ESR1**	GGGAAGTATGGCTATGGAATCTG	TGGCTGGACACATATAGTCGTT
**AR**	TACCAGCTCACCAAGCTCCT-	GCTTCACTGGGTGTGGAAAT
**CCNB1**	GGTTGGGTCGGCCTCTACCT	AGCCAGGTGCTGCATAACTGGAA
**CDK1**	CGTGGGGGAGCGGATTT	CGGAGGGCGAGTATTGAGGA
**AKR1C3**	GTTGCCTATAGTGCTCTGGGATCT	GGACTGGGTCCTCCAAGAGG
**CCNA2**	GTAAACAGCCTGCGTTCACC	ACTTCAACTAACCAGTCCACGAG
**GAPDH**	ACCACAGTCCATGCCATCAC	TCCACCACCCTGTTGCTGTA

### Cell Counting Kit−8 (CCK−8) Assay to Detect Cell Proliferation

CCK−8 assay was performed to detect cell viability and cell proliferation. The HepG2 cells and Huh7 cells were seeded in the 96-well culture plates at a density of 1×10^5^ cells/well and incubated for 24 h at 37°C and 5% CO2 atmosphere, respectively. Cells were exposed to 200 mg/L PADP and further incubated for 24 h. The medium was replaced with 100 µL of fresh medium containing 10% CCK8 (Dojindo Molecular Technologies, Inc), and cells were incubated for 4 h at 37°C and 5% CO2 atmosphere. The OD450 nm absorbance value in each well was determined by the scanning porous spectrophotometer (Thermo Scientific, China). Cell proliferation rate (%) was used to describe the effect of PADP on cellular viability, and calculated as follow equation: Cell proliferation rate (%) = (OD PADP – OD Blank)/(OD Control – OD Blank)× 100%.

### Flow Cytometry Detect the Cell Cycle Distribution and Apoptotic Rate

Flow cytometry was conducted to detect the cell cycle distribution and apoptotic rate. The Annexin V−FITC/PI cell apoptosis detection kit and cell cycle assay kits were bought from NanJing KeyGen Biotech Co., Ltd. (cat. no. KGA108). Cells were digested with 0.25% pancreatin (MedChemExpress; cat. no. HY−B2118) without EDTA. Cell apoptosis was assessed using the AnnexinV−FITC/PI cell apoptosis detection kit according to the manufacturer’s protocol. Briefly, cells were re−suspended in 500 µl binding buffer mixed with 5 µl AnnexinV−FITC, then mixed with 5 µl PI and incubated at room temperature in the dark for 15 min. The cell cycle was assessed using the cell cycle detection kit according to the manufacturer’s protocol. Cells were washed with PBS, centri−fuged with 350 x g for 5 min at 4°C, fixed with pre−cooled 70% ethanol at 4°C for 1−2 h, washed for a second time and the cell suspension was stained at 37°C for 15 min with 1 ml PI/Triton X−100 (20 µg PI/0.1% Triton X−100) containing 0.2 mg RNase.

### Transwell Invasion Assay to Detect Cell Invasion

Transwell invasion assay was performed to detect cell invasion. After adjusted at the concentration of 1 × 10^5^ cells/mL with the medium, The HepG2 cells and Huh7 cells were seeded 100 µL/well into the upper chamber of the transwell migration chamber. The medium with a concentration of 200mg/L of PADP was added lower chamber. The cells were then incubated for 24 h at 37°C and 5% CO2 atmosphere. The chamber was removed and fixed with methanol for 20 min. After dried at room temperature, the cells were stained with crystal violet for 20 min. Cells that did not pass through the upper part of the chamber were removed with a wet cotton swab, and then the chamber was placed under an inverted microscope to calculate remaining cells.

### Statistical Analysis

SPSS 13.0 software was used for statistical analysis of the data. The results were expressed as means ± SDs. One-way analysis of variance (ANOVA) or Student’s t test was performed to examine the differences between groups. A *P* value less than 0.05 was considered statistically significant.

## Results

### GEO Gene Chip and Differential Gene Analysis

**Figure 1:**
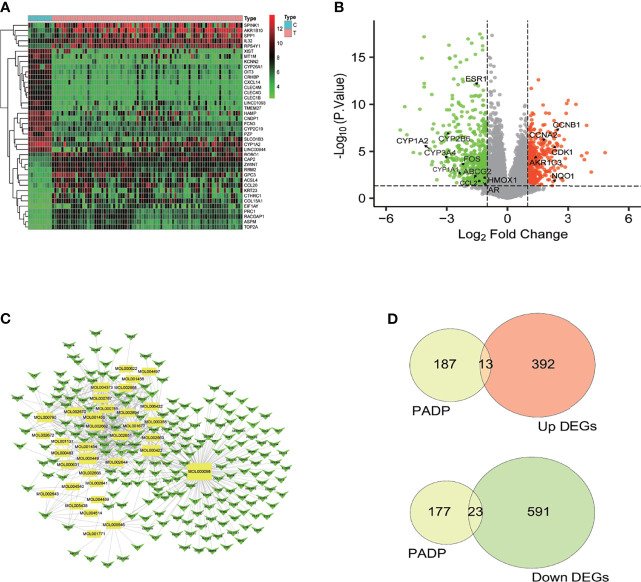
The volcano and heat map of DEGs, compounds-targets network diagram and differential genes of liver cancer by PADP. **(A)** In the heat map, the abscissa represents the sample name, the ordinate represents the gene name, the light blue part represents the normal sample, and the pink part represents the tumor sample. The green part represents the low expression of the gene in the sample, the red part represents the high expression, and the black part is in between. **(B)** In the volcano diagram, the green part represents genes that are up-regulated in the normal group, the red part represents genes that are up-regulated in the tumor group, and the black parts represent genes that have no difference between the normal group and the tumor group. **(C)** The yellow rectangular nodes represent compounds, and the green arrows represent genes. The larger the graph area, the more adjacent nodes that represent the node, and the more important the node is. **(D)** Intersection of up-regulated and down-regulated differential genes of liver cancer by PADP.

A total of 21654 genes were obtained by analyzing the GSE62232 gene chip. After screening, a total of 1019 differential genes were obtained, including 614 down-regulated genes and 405 up-regulated genes. The heat map and volcano diagram of the differential genes were shown in **Figures 1A, B** respectively.

### Drug Chemical Components and Targets

After searching the TCMSP database and screening by the ADME standard, a total of 221 compounds and 52 active ingredients were obtained, of which Phellodendron had 140 compounds and 37 active ingredients, while Anemarrhena had 81 compounds and 15 active ingredients. The target genes corresponding to the above active ingredients were combined and deduplicated, and 201 target genes were finally obtained. Compounds-targets network diagram was shown in **Figure 1C**. The annotations for active ingredients and targets was shown in **Table S1** in Supplementary Material. The top 10 core compounds selected according to **Figure 1C** and **Table S1** were shown in **Table 2**.

**Table 2:** Core compounds.

No.	Degree layout	Mol ID	Compound	2D structure	OB	DL	Herb
1	141	MOL000098	quercetin	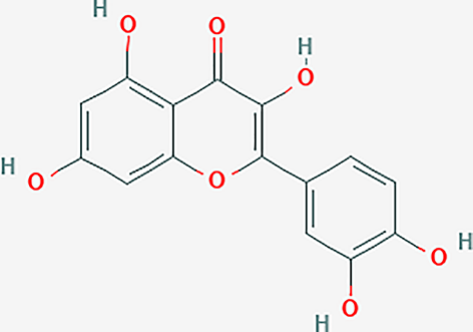	46.43	0.28	Phellodendron
2	56	MOL000422	kaempferol	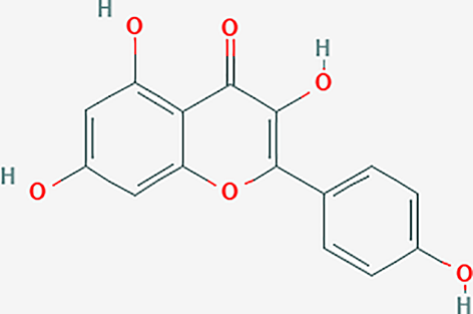	41.88	0.24	Anemarrhena
3	54	MOL000449	stigmasterol	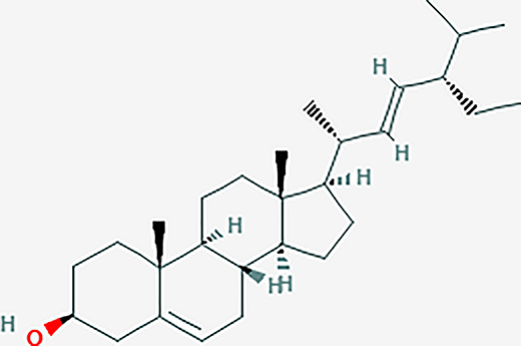	43.83	0.76	Phellodendron
4	32	MOL004373	Anhydroicaritin	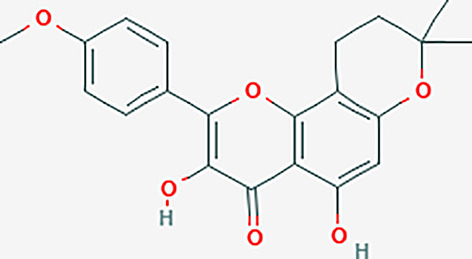	45.41	0.44	Phellodendron
5	31	MOL000790	Isocorypalmine	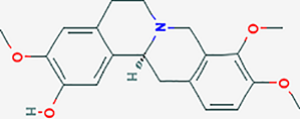	35.77	0.59	Anemarrhena
6	28	MOL000358	beta-sitosterol	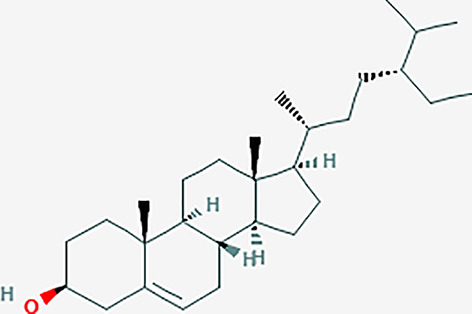	36.91	0.75	Phellodendron
7	28	MOL001455	(S)-Canadine	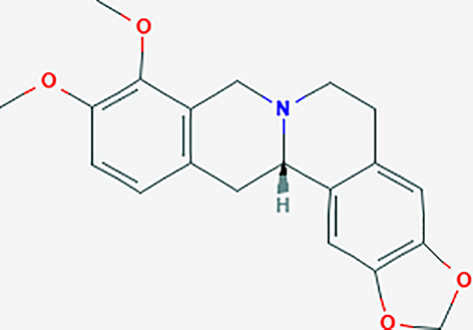	53.83	0.77	Phellodendron
8	24	MOL002670	Cavidine	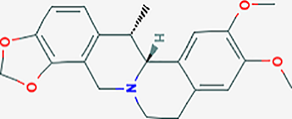	35.64	0.81	Phellodendron
9	22	MOL000787	Fumarine	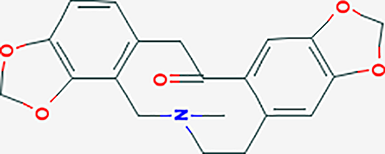	59.26	0.83	Phellodendron
10	19	MOL002651	Dehydrotanshinone II A	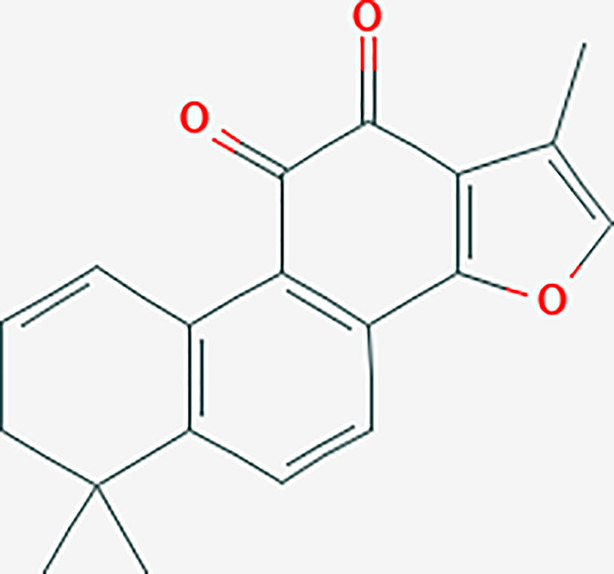	43.76	0.4	Phellodendron

### Intersection of Disease Genes and Drug Genes

The up-regulated genes and down-regulated genes in the liver cancer differential genes were intersected with the genes of PADP to obtain the Venn diagram. As can be seen from **Figure 1D**, there were 200 target genes for the active ingredient predicted by PADP, and 405 genes were up-regulated, and 614 differential genes were down-regulated in liver cancer. There were 36 genes of PADP for the treatment of liver cancer, of which 13 genes were up-regulated and 23 genes were down-regulated (**Figure 1D**). The up-regulated genes included BAX, MAP2, CDK1, BIRC5, HSPB1, CCNB1, TOP2A, NQO1, CXCL11, CXCL10, SPP1, AKR1C3 and CCNA2. While the down-regulated genes included ESR1, AR, ADRB2, ADRA1A, CYP3A4, CYP1A2, CYP2B6, NR3C2, ADH1C, ADRB1, PON1, CA2, FOS, HMOX1, CYP1A1, CCL2, SULT1E1, NR1I2, ABCG2, CXCL2, NR1I3, IGFBP3 and PCOLCE.

### Construction of Traditional Chinese Medicine Compound Regulation Network

**Figure 2:**
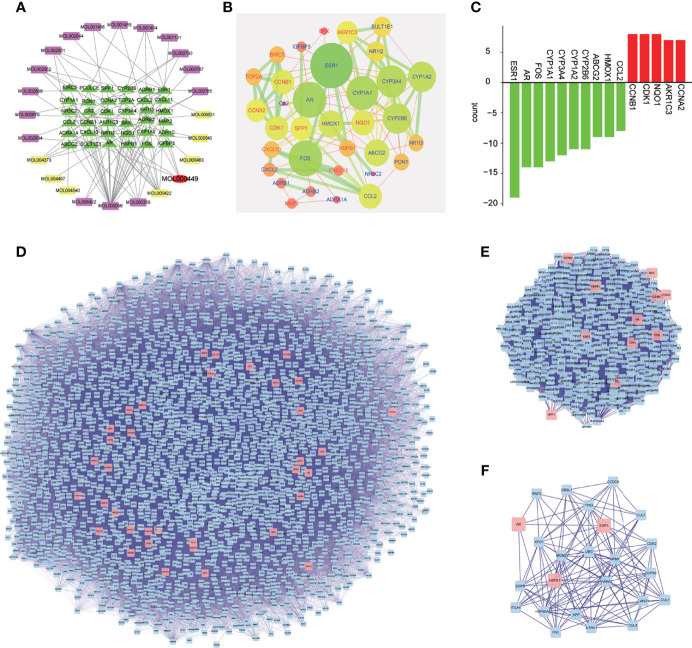
Chinese medicine compound regulation network, core genes, and analysis of PPI and network topology. **(A)** Purple represents the active ingredient of Phellodendron, yellow represents the active ingredient of Anemarrhena, red is the active ingredient shared by both, green is the target gene corresponding to the active ingredient. **(B)** The nodes represent genes. The larger the nodes, the more important genes are. The color scale changes from red to green, and the closer the color is to green, the more important the gene is. The node label red represents high-expressing genes and blue represents low-expressing genes. Besides, the line indicates the strength of the association between genes, and the thicker the line, the stronger the degree of association. **(C)** The abscissa represents the gene name, red represents up-regulated genes while green represents down-regulated genes, and the ordinate represents the number of genes. **(D–F)** Analysis of PPI and network topology.

The traditional Chinese medicine compound regulation network diagram consisted of 61 nodes. It can be seen from **Figure 2A** that there were 17 effective components of Phellodendron and 8 effective components of Anemarrhena for the treatment of liver cancer. And MOL000449 was an effective component for treating liver cancer shared by both. Besides, there were 36 genes of PADP for treating liver cancer.

### Screening of Core Genes

The PPI network diagram of PADP for liver cancer was shown in **Figure 2B**. A bar graph of the first 10 down-regulated genes and the first 5 up-regulated genes was shown in **Figure 2C**.

### Construction of PPI Network and Analysis of Network Topology

The gene of PADP for treating liver cancer was input into Cytoscape software, and the PPI network diagram was obtained using the “Bisogenet” package (**Figure 2D**). The nodes represent proteins, and the links represent the degree of association between proteins. It can be seen from **Figure 2D** that the PPI network had a total of 2352 nodes and 57734 connections. The first filter, set degree > 81, and finally got 414 nodes and 16688 connections (**Figure 2E**). The second filter, set betweeness > 200, and finally got 24 nodes and 140 connections (**Figure 2F**).

### GO Biological Process and KEGG Pathway Enrichment Analysis

**Figure 3:**
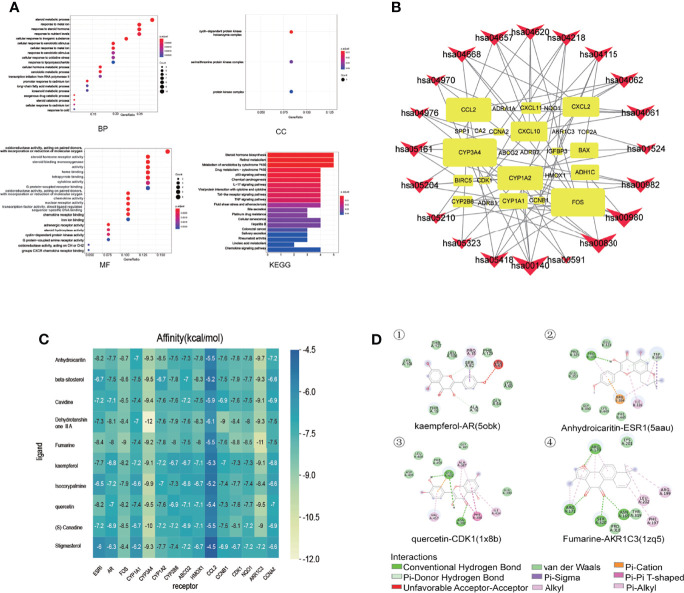
GO biological process and KEGG pathway enrichment analysis, and molecular docking of core compounds and core target genes. **(A)** The abscissa of the GO biological process bubble chart represents the proportion of genes, and the ordinate represents the names of enriched biological processes, cell components, and molecular functions, respectively. The bubble size represents the proportion of genes on each biological process. The larger the bubble, the more genes are enriched. The horizontal axis of the KEGG pathway bubble chart represents the proportion of the pathway in the total pathway, and the vertical axis represents the pathway name. The bar length represents the proportion of genes on each pathway. The longer the bar, the more genes are enriched. The color represents the degree of enrichment. And the closer the color is to red, the more significant the enrichment is. **(B)** KEGG relationship network construction. In the KEGG relationship network diagram, the red arrows represent pathways, the yellow rectangles represent genes, and the links represent the relationship between pathways and genes. The larger the red arrow, the greater the number of genes enriched in the pathway. Similarly, the larger the yellow rectangle, the more pathways connected to the gene. **(C)** The abscissa represents the core gene, and the ordinate represents the core compound. The numbers in the box represent the docking affinity score. The grading color scale ranges from blue to yellow. The closer the color is to yellow, the lower the affinity score is and the stronger the binding is. **(D)** The figure is the two-dimensional structure diagram of ligand and receptor interaction. Different color represents different interaction.

The R language was used to perform enrichment analysis of GO biological process and KEGG pathway for treatment of liver cancer with PADP. Finally, the enrichment numbers of BP, CC, and MF were 397, 3, and 44, respectively. A total of 23 pathways were related to the efficacy of PADP for the treatment of liver cancer. The bubble chart of the top 20 entries was shown in **Figure 3A**.

### KEGG Relationship Network Construction

A KEGG relationship network diagram was constructed for the top 20 pathways and genes enriched in pathways for the treatment of liver cancer by PADP (**Figure 3B**). It can be seen from **Figure 3B** that genes such as CYP3A4, CYP1A2, FOS, CCL2, CXCL2, CXCL10, CYP1A1, ADH1C, BAX, and CYP2B6 occupied a large rectangular area, indicating that these genes play a key role in the mechanism of PADP on the treatment of liver cancer.

### Molecular Docking Verification of Core Compounds and Core Target Genes

The protein crystals corresponding to the core target genes were searched through the PDB database, and the protein crystal names corresponding to the up-regulated genes CCNB1, CDK1, NQO1, AKR1C3, CCNA2 were 6guk, 1x8b, 1h69, 1zq5 and 6gue. While the protein crystal names corresponding to the down-regulated genes ESR1, AR, FOS, CYP1A1, CYP3A4, CYP1A2, CYP2B6, ABCG2, HMOX1 and CCL2 were 5aau, 5obk, 1kms, 6o5y, 4d6z, 2hi4, 5ufg, 5tf7, 6qgv and 5coy, respectively. The docking results were shown in **Figure 3C**. It can be seen from **Figure 3C** that except for the corresponding crystal affinity scores of stigmasterol and CCL2 of -4.5 kcal/mol, the scores of the other compounds and protein crystals were all less than -5 kcal/mol, suggesting that the above-mentioned core compounds have good affinity with core gene proteins. Part of the interaction forces between ligands and receptors were shown in **Figure 3D**.

### Survival Analyses for Hub Genes

**Figure 4:**
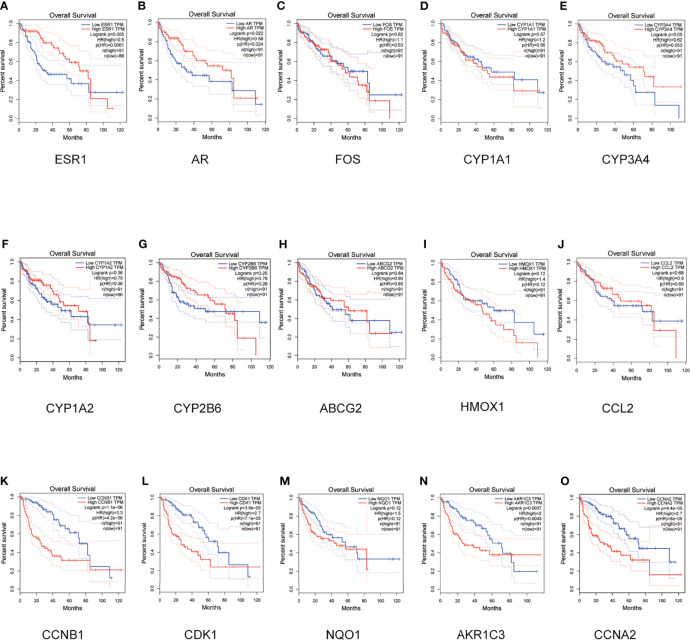
Survival analyses for hub genes. OS of the 10 down-regulated and 5 up-regulated hub genes was analyzed by Kaplan-Meier plotter. Data are presented as the hazard ratio with a 95% confidence interval. The log-rank P of the down-regulated hub genes ESR1 **(A)**, AR **(B)**, FOS **(C)**, CYP1A1 **(D)**, CYP3A4 **(E)**, CYP1A2 **(F)**, CYP2B6 **(G)**, ABCG2 **(H)**, HMDX1 **(I)** and CCL2 **(J)** were 0.005, 0.022, 0.62, 0.57, 0.05, 0.36, 0.25, 0.84, 0.12 and 0.68. The log-rank P of the up-regulated hub genes CCNB1 **(K)**, CDK1 **(L)**, NQO1 **(M)**, AKR1C3 **(N)** and CCNA2 **(O)** were 1.1e-06, 3.9e-05, 0.12, 0.0037 and 4.4e-05. Log-rank *P* < 0.05 was regarded as statistically significant.

As shown in **Figure 4**, GEPIA online analysis tool was used to perform survival analysis of the 5 up-regulated and 10 down-regulated genes. The survival analysis of down-regulated genes ESR1 and AR, up-regulated genes CCNB1, CDK1, AKR1C3 and CCNA2 is statistically significant (P<0.05). Therefore, key targets ESR1, AR, CCNB1, CDK1, AKR1C3 and CCNA2 were identified for experimental verification.

### Changes of the mRNA of the Key Target Genes and the Validation of Cell Proliferation, Apoptotic, Cell Cycle and Invasion

**Figure 5:**
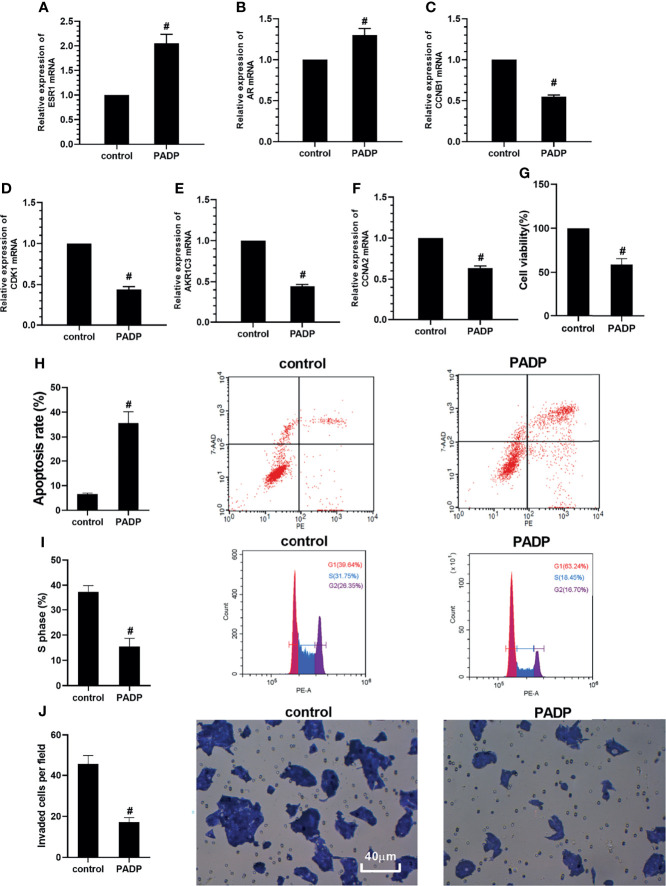
Effect of PADP on the mRNA expression of key target genes and on the cell proliferation, apoptotic and cell cycle and invasion of HepG2 cells. Effect of PADP on the mRNA expression of ESR1 **(A)**, AR **(B)**, CCNB1 **(C)**, CDK1 **(D)**, AKR1C3 **(E)** and CCNA2 **(F)**. CCK−8 assay was performed to detect cell viability **(G)**. Flow cytometry was performed to detect the apoptotic rate **(H)** and proportion of S phase cells **(I)**. Transwell invasion assay was performed to detect cell invasion **(J)**. Values are expressed as mean ± SD. ^#^ *P* < 0 05, compared with control group.

RT-PCR was used to verify the effect of PADP on the changes of target genes in human hepatocarcinoma HepG2 cell line. As shown in **Figures 5A–F**, compared with the control group, the mRNA expression of ESR1 and AR were increased in PADP group (*P*<0.05), and the mRNA expression of CCNB1, CDK1, AKR1C3 and CCNA2 were decreased in PADP group (*P*<0.05). The effect of PADP on cell proliferation of HepG2 cells was measured using the CCK-8 assay. As shown in **Figure 5G**, the cell viability rate of HepG2 cells decreased significantly after administered of PADP (*P*<0.05). The effect of PADP on cell apoptotic and cell cycle of HepG2 cells were measured using the Flow cytometry. As shown in **Figures 5H, I**, the apoptotic rate was significantly increased in PADP group (*P*<0.05), and proportion of S phase cells was significantly decreased in PADP group (*P*<0.05). Transwell invasion assay was performed to detect cell invasion. As shown in **Figure 5J**, the invaded cells per field were significantly decreased in PADP group (*P*<0.05). Likewise, as shown in **Figure S1** in the Supplementary Material, we came to similar conclusions in the Huh7 cell line. The above cell-based assays showed that PADP could promote the apoptosis of liver cancer cells, shorten the cell cycle, and inhibit the proliferation and invasion of liver cancer cells.

## Discussion

Liver cancer is one of the most widespread types of cancer and is notorious for its high morbidity and mortality (21, 22). In this study, bioinformatics technology was used to obtain differential genes of liver cancer. At the same time, network pharmacology technology was used to obtain the effective components of Phellodendron and Anemarrhena and corresponding target genes, which provided important theoretical basis for the treatment of liver cancer with PADP.

Firstly, in this study, the GSE62232 chip was analyzed by using bioinformatics technology. A total of 1019 differential genes were obtained, including 614 down-regulated genes and 405 up-regulated genes. After searching the TCMSP database and screening by ADME standards through Chinese medicine network pharmacology technology, a total of 52 active ingredients of PADP were obtained, of which 37 active ingredients were Phellodendron and 15 active ingredients were Anemarrhena.

After intersecting the disease differential genes with the drug target genes, there were 36 genes from PADP to treat liver cancer, of which 13 genes were up-regulated and 23 genes were down-regulated. The above-mentioned differential genes and predicted active ingredient targets were used to construct a traditional Chinese medicine active ingredients-targets network diagram of PADP in the treatment of liver cancer. It can be seen from **Figure 2A** that the number of effective components of Phellodendron and Anemarrhena for treating liver cancer were 17 and 8 respectively, of which MOL000449 (stigmasterol) was shared by both. The main target genes of this effective component included NR3C2, ADH1C, ADRB2, ADRB1, ADRA1A. Studies have shown that plant sterols such as stigmasterol have tumor suppressive effects and can reduce beta amyloid production through different mechanisms (23, 24).

Secondly, this study performed PPI analysis on the above target genes. It is well known that protein-protein interactions are the basis of cellular functions in living organisms and play an important role in regulating physiological and pathological conditions. The core genes of PPI were further studied to obtain the core genes network diagram and bar graph. Simultaneously, the PPI and network topology analysis were performed on the genes of PADP for treating liver cancer. From the topological structure of the PPI network in **Figures 2D–F**, in addition to the three genes (ESRI, AR, and HSPB1) predicted above, the PADP may be related to many other genes in the treatment of liver cancer. After screening, 21 genes highly related to the treatment of liver cancer by PADP were finally obtained, including TP53, NTRK1, CUL3, CDK2, MCM2, CUL7, and COPS5. Among them, TP53, MCM2, and CDK2 are mainly related to the cell cycle. Studies have shown that TP53 is an important tumor suppressor, and about 30-50% of mutations occur in liver cancer (25). MCM2 is significantly related to the survival and progression of liver cancer, and its potential mechanism in liver cancer prognosis may involve the cell cycle (26). CDK2 is expressed in hepatocellular carcinoma stem cells and can promote the cycle progression of liver cancer cells (27). CUL3 and CUL7 are members of the Cullin ubiquitin ligase family and are involved in the regulation of various cancer-related biological pathways. Liu G (28) showed that CUL7 can promote epithelial-mesenchymal transition in liver cancer, and its high expression in liver tumors is related to poor prognosis. Besides, COPS5 can regulate the deubiquitination of cullin, thereby controlling a variety of biological processes, which has been reported in a variety of cancers (liver, pancreatic, breast, etc) (29).

Meanwhile, this study used R language to perform GO biological process enrichment analysis and KEGG pathway analysis of PADP for treating liver cancer genes. From the analysis of GO biological process enrichment, it can be seen that BP mainly focused on life processes such as metabolism, cellular response, and transcription; CC mainly focused on cell components of protein kinase complexes; MF mainly focused on the aspects of enzyme activity and the binding reaction of various biological processes. It can be seen from **Figure 3A** that the PADP can play a role in treating liver cancer through multiple pathways, including p53 signaling pathway, IL-17 signaling pathway, TNF signaling pathway, Toll-like receptor signaling pathway and Chemokine signaling pathway. In addition, it can be known from the signaling pathway that the diseases most closely related to liver cancer were hepatitis B colorectal cancer. The macro level of the signaling pathways mainly promotes the occurrence of liver cancer by affecting bile secretion, endocrine resistance, platinum drug resistance, chemical carcinogenesis. And at the micro level, it mainly affects cellular senescence, apoptosis-multiple species, biosynthesis and metabolism of cytochrome P450 and hormone, and then promotes the occurrence and development of liver cancer.

At the same time, a KEGG relationship network was constructed for the KEGG enrichment pathway and core genes in this study. In addition to the above signaling pathways, there were numerous biological processes that affect the occurrence and progression of liver cancer, among which hsa00980 (Metabolism of xenobiotics by cytochrome P450), hsa0083 (Retinol metabolism) and hsa00140 (Steroid hormone biosynthesis) had the most abundant genes. As we all know, the liver is known to be the central organ regulating glucose homeostasis, exogenous metabolism, and steroid biosynthesis and degradation (30). Steroid hormone biosynthesis is mainly achieved by enzymes of the cytochrome P450 family (31). Metabolism of xenobiotics by cytochrome P450 is a group of membrane-bound proteins that are mainly found in the liver and intestine. It can catalyze the metabolism of a variety of endogenous and exogenous substances, and is closely related to the occurrence of liver cancer (32–34). The liver is the most important storage organ, which contains retinol metabolizing enzymes and participates in retinol metabolism (35). Researches have shown that decreased hepatic retinol levels were observed in chemically induced liver injury, suggesting retinol metabolism were increased in liver diseases (36). Studies by Cheng YW et al. showed that retinol metabolism was involved in the process of liver fibrosis and was closely related to malignant tumors (37). In addition, it can be inferred from **Figure 3B** that genes such as CYP3A4, CYP1A2, FOS, CCL2, CXCL2, CXCL10, CYP1A1, ADH1C, BAX, and CYP2B6 play a key role in the mechanism of PADP on the treatment of liver cancer. Among them, CYP3A4 (38), CYP1A2 (39), CYP1A1 (40) and CYP2B6 (41) are members of the cytochrome P450 family and they are involved in the pathogenesis of liver cancer in certain ways. Increasing evidence indicate that chemokines and their receptors play a role in tumorigenesis, development and metastasis. CXCL2 and CXCL10 are genes enriched by Chemokine signaling pathway; CXCL2 promotes the proliferation and metastasis of liver cancer cells (42); and CXCL10 is associated with enhanced T cell infiltration in tumors (43). In addition, genes such as FOS (44), CCL2 (45), ADH1C (46) and BAX (47) are also associated with the occurrence of liver cancer.

Finally, according to the molecular docking results in **Figure 3C**, it can be seen that the small molecule compound was tightly bound to the protein residue through various interaction forces such as van der waals, convational hydrogen bond, pi-sigma, pi-pi T-shaped, pi-cation, pi-donor hydrogen bond and so on. It can be seen from **Figure 4** that the survival analysis of down-regulated genes such as ESR1 and AR, up-regulated genes such as CCNB1, CDK1, AKR1C3 and CCNA2 is statistically significant, suggesting that the above genes may become potential target genes for the survival and prognosis of PADP for the treatment of liver cancer. RT-PCR indicated that PADP can increase the mRNA expression of ESR1 and AR and decrease the mRNA expression of CCNB1, CDK1, AKR1C3 and CCNA2. Meanwhile, cell assays showed that PADP could promote the apoptosis of liver cancer cells, shorten the cell cycle, and inhibit the proliferation and invasion of liver cancer cells. ERS1 and AR were identified as relatively high-involved molecules, which suggested that these proteins may play essential roles in HCC progression (48). The polymorphisms of ESR1 were related to HCC risk among chronic HBV carriers (49). AR plays critical roles in HCC, it is one of the important target molecules for the treatment of HCC (50). AR might enhance HCC initiation and eary development, but suppress HCC metastasis at the later stages of the disease (51). Studies have shown that high expression of CDK1, CCNB1, and CCNA2 is associated with reduced overall survival in patients with liver cancer (52). CCNB1 was related with cell proliferation in HCC patients. In addition, CCNB1 is also closely related to the proliferation, migration and invasion of hepatoma cells (53). CDK1 plays an important regulatory role in cell cycle regulation, and CDK1 can increase cellular viability and promote proliferation in HCC cell lines (54). AKR1C3 is known to be involved in the metabolism and biosynthesis of estrogens, androgens, progesterone, and prostaglandins. AKR1C3 can promote tumor cell proliferation, metastasis, invasion, and angiogenesis, and inhibit tumor cell apoptosis and differentiation (55). The upregulation of CCNA2 might thus play a key role in the dysregulation of normal growth in HCC carcinogenesis (56).

**Figure 6:**
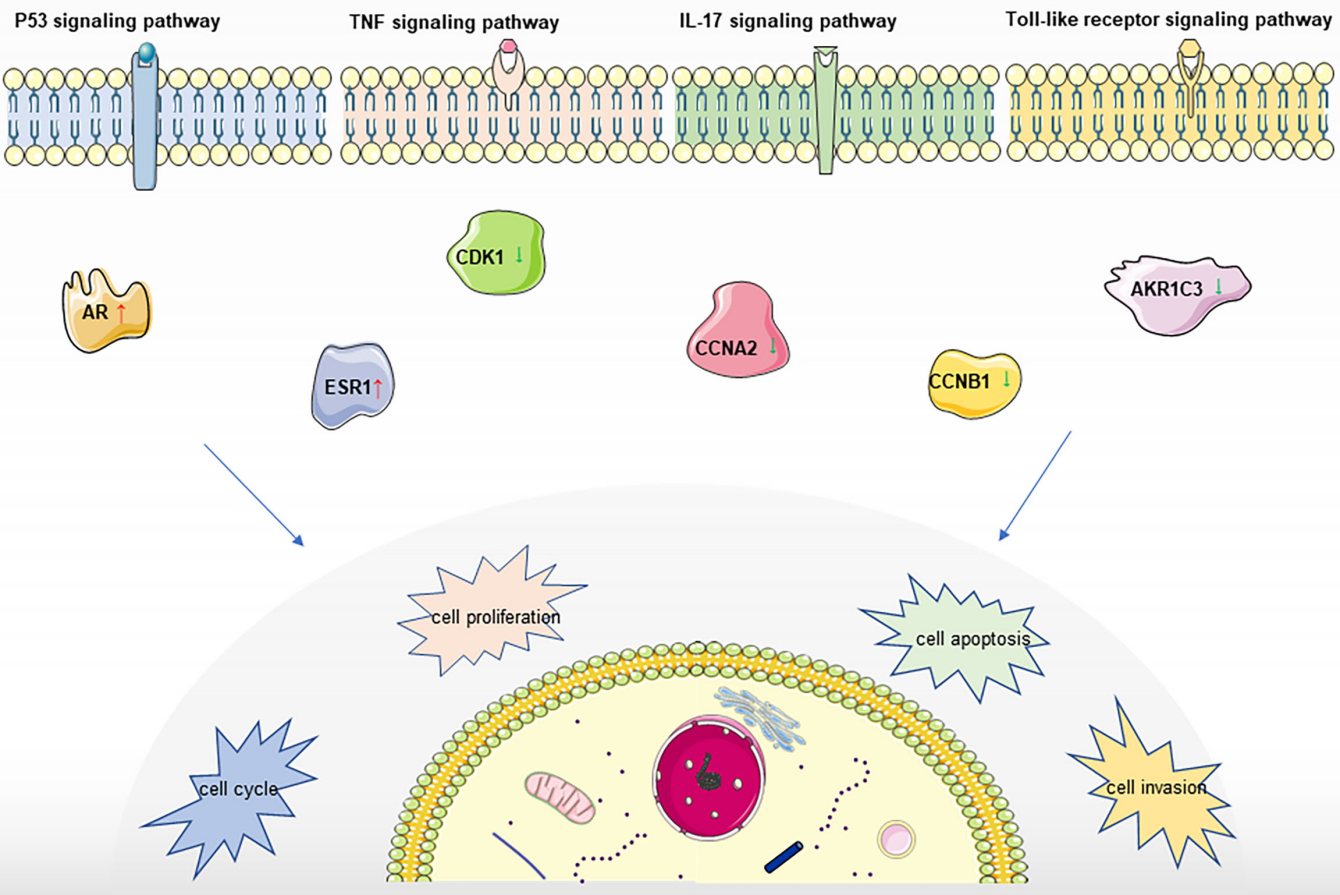
Mechanism of PADP in the treatment of liver cancer.

In summary, PADP may up regulate genes ESR1, AR, and down regulate genes CCNB1, CDK1, AKR1C3, and CCNA2, through p53 signaling pathway, IL-17 signaling pathway, TNF signaling pathway and Toll-like receptor signaling pathway, thereby promoting the apoptosis of liver cancer cells, shortening the cell cycle, and inhibiting the proliferation and invasion of liver cancer cells. And the mechanism of action is shown in **Figure 6**.

## Data Availability Statement

The datasets presented in this study can be found in online repositories. The names of the repository/repositories and accession number(s) can be found in the article/**Supplementary Material**.

## Author Contributions

XR and YW conceived and designed the study. XR and YW wrote the paper. XR, WL, and PD performed the study and analyzed the data. YW and PD supervised the study and revised the manuscript. All authors read and approved the final manuscript.

## Funding

This work was supported by the National Science Foundation of China (82100630 and 82100894) and by the Fundamental Research Funds for the Central Universities (2042021kf0080).

## Conflict of Interest

The authors declare that the research was conducted in the absence of any commercial or financial relationships that could be construed as a potential conflict of interest.

## Publisher’s Note

All claims expressed in this article are solely those of the authors and do not necessarily represent those of their affiliated organizations, or those of the publisher, the editors and the reviewers. Any product that may be evaluated in this article, or claim that may be made by its manufacturer, is not guaranteed or endorsed by the publisher.

## Abbreviations

BP, Biological process; CC, Cellular component; DAVID, Database for Annotation, Visualization and Integrated Discovery; DL, Drug-like; GEO, Gene Expression Omnibus; GO, Gene Ontology; KEGG, Kyoto Encyclopedia of Genes and Genomes; MF, Molecular function; OB, Oral bioavailability; OS, Overall Survival; PADP, Phellodendron and Anemarrhena drug pair; PDB, Protein Data Bank; PPI, Protein-protein interaction; STRING, Search Tool for the Retrieval of Interacting Genes; TCMSP, Traditional Chinese Medicine Systems Pharmacology Database and Analysis Platform; UniProt, Unified Protein; CCK−8, Cell Counting Kit−8.
