# Supplementary material for: Mechanism of Phellodendron and Anemarrhena Drug Pair on the Treatment of Liver Cancer Based on Network Pharmacology and Bioinformatics
Source: Front Oncol. 2022 Apr 7;12:838152. doi: 10.3389/fonc.2022.838152 (PMC9021729; doi:10.3389/fonc.2022.838152)
Supplement: Supplementary file 1 [file DataSheet_1.docx]

**Supplementary information**

**Figure legends for Figure S1** Effect of PADP on the mRNA expression of core genes and on the cell proliferation, apoptotic and cell cycle and invasion of Huh7 cells. Effect of PADP on the mRNA expression of ESR1 (A), AR (B), CCNB1 (C), CDK1 (D), AKR1C3 (E) and CCNA2 (F). (G) CCK‑8 assay was performed to detect cell viability. (I) Flow cytometry was performed to detect the apoptotic rate (H) and proportion of S phase cells. (J) Transwell invasion assay was performed to detect cell invasion. Values are expressed as mean ± SD. #*P* < 0 05, compared with control group.


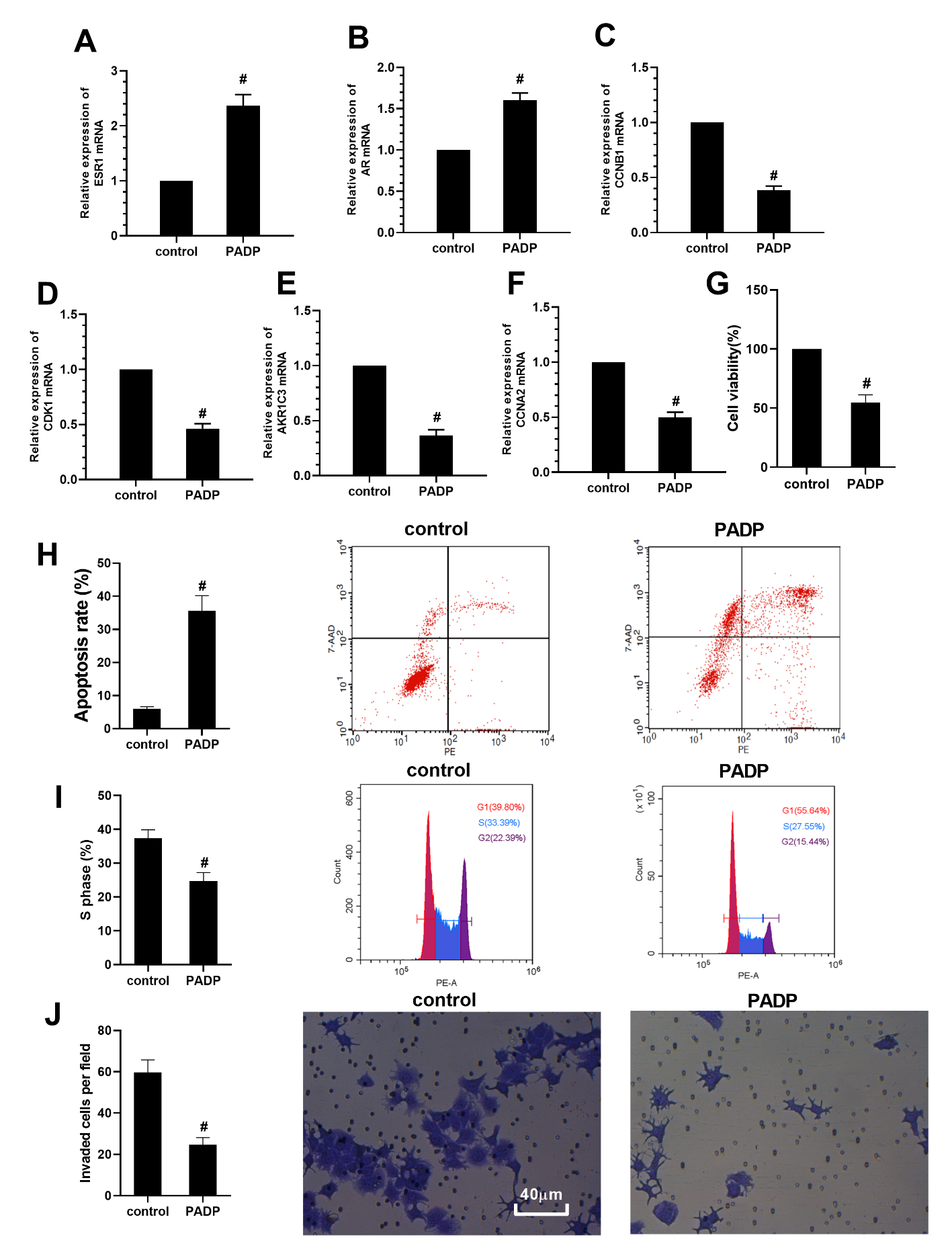


**Table S1 Drugs-targets network**

| degree.layout | name | type | degree.layout | name | type |
| --- | --- | --- | --- | --- | --- |
| 141 | MOL000098 | mollist | 2 | ALOX5 | genelist |
| 56 | MOL000422 | mollist | 2 | HAS2 | genelist |
| 54 | MOL000449 | mollist | 2 | GSTP1 | genelist |
| 32 | MOL004373 | mollist | 2 | AHR | genelist |
| 31 | MOL000790 | mollist | 2 | PSMD3 | genelist |
| 28 | MOL000358 | mollist | 2 | SLC2A4 | genelist |
| 28 | MOL001455 | mollist | 2 | NR1I3 | genelist |
| 26 | PTGS2 | genelist | 2 | INSRR | genelist |
| 24 | MOL002670 | mollist | 2 | DIO1 | genelist |
| 22 | MOL000787 | mollist | 2 | GSTM1 | genelist |
| 20 | PTGS1 | genelist | 2 | GSTM2 | genelist |
| 19 | MOL002651 | mollist | 2 | MOL001771 | mollist |
| 18 | SCN5A | genelist | 2 | MOL004514 | mollist |
| 18 | HSP90AA1 | genelist | 1 | MOL002643 | mollist |
| 16 | MOL000785 | mollist | 1 | IL4 | genelist |
| 16 | MOL000546 | mollist | 1 | CYP2B6 | genelist |
| 15 | RXRA | genelist | 1 | MOL002672 | mollist |
| 15 | ADRB2 | genelist | 1 | CHRNA2 | genelist |
| 14 | MOL001454 | mollist | 1 | MAP2 | genelist |
| 14 | KCNH2 | genelist | 1 | GRIA2 | genelist |
| 14 | MOL002662 | mollist | 1 | CACNA1S | genelist |
| 13 | NCOA2 | genelist | 1 | CA2 | genelist |
| 13 | CHRM1 | genelist | 1 | MMP3 | genelist |
| 12 | AR | genelist | 1 | EGFR | genelist |
| 12 | CALM1 | genelist | 1 | CCND1 | genelist |
| 12 | ADRA1B | genelist | 1 | BCL2L1 | genelist |
| 12 | MOL006422 | mollist | 1 | FOS | genelist |
| 11 | MOL002894 | mollist | 1 | EIF6 | genelist |
| 10 | PGR | genelist | 1 | MAPK1 | genelist |
| 10 | MOL002644 | mollist | 1 | IL10RB | genelist |
| 10 | GABRA1 | genelist | 1 | EGF | genelist |
| 10 | CHRM3 | genelist | 1 | RB1 | genelist |
| 9 | ESR1 | genelist | 1 | IL6 | genelist |
| 9 | PRSS1 | genelist | 1 | ELK1 | genelist |
| 9 | F7 | genelist | 1 | NFKBIA | genelist |
| 9 | MOL004497 | mollist | 1 | POR | genelist |
| 9 | MOL000631 | mollist | 1 | ODC1 | genelist |
| 8 | NOS2 | genelist | 1 | TOP1 | genelist |
| 8 | MOL001458 | mollist | 1 | RAF1 | genelist |
| 7 | HTR | genelist | 1 | RUNX1T1 | genelist |
| 7 | OPRM1 | genelist | 1 | HSPA5 | genelist |
| 7 | MOL001131 | mollist | 1 | ERBB2 | genelist |
| 7 | MOL000483 | mollist | 1 | ACACA | genelist |
| 6 | DRD1 | genelist | 1 | CAV1 | genelist |
| 6 | PPARG | genelist | 1 | MYC | genelist |
| 6 | CHRM5 | genelist | 1 | F3 | genelist |
| 6 | CHRM4 | genelist | 1 | GJA1 | genelist |
| 6 | OPRD1 | genelist | 1 | IL1B | genelist |
| 6 | ADRA1A | genelist | 1 | CCL2 | genelist |
| 6 | CHRM2 | genelist | 1 | PTGER3 | genelist |
| 6 | MOL002668 | mollist | 1 | IL8RA | genelist |
| 5 | DPP4 | genelist | 1 | PRKCB | genelist |
| 5 | NCOA1 | genelist | 1 | BIRC5 | genelist |
| 5 | HTR3A | genelist | 1 | DUOX2 | genelist |
| 5 | MOL002666 | mollist | 1 | NOS3 | genelist |
| 5 | SLC6A3 | genelist | 1 | HSPB1 | genelist |
| 5 | SLC6A4 | genelist | 1 | SULT1E1 | genelist |
| 4 | ACHE | genelist | 1 | MGAM | genelist |
| 4 | NR3C2 | genelist | 1 | IL2 | genelist |
| 4 | IGHG1 | genelist | 1 | CCNB1 | genelist |
| 4 | SLC6A2 | genelist | 1 | PLAT | genelist |
| 4 | LTA4H | genelist | 1 | THBD | genelist |
| 4 | MAOB | genelist | 1 | SERPINE1 | genelist |
| 4 | ADRB1 | genelist | 1 | COL1A1 | genelist |
| 4 | ADRA1D | genelist | 1 | IFNGR1 | genelist |
| 4 | RXRB | genelist | 1 | IL1A | genelist |
| 4 | NR3C1 | genelist | 1 | MPO | genelist |
| 4 | MOL001677 | mollist | 1 | TOP2A | genelist |
| 3 | MOL002641 | mollist | 1 | NCF1 | genelist |
| 3 | CHEK1 | genelist | 1 | ABCG2 | genelist |
| 3 | TNFSF15 | genelist | 1 | NFE2L2 | genelist |
| 3 | CYP3A4 | genelist | 1 | NQO1 | genelist |
| 3 | CYP1A2 | genelist | 1 | PARP1 | genelist |
| 3 | MOL002663 | mollist | 1 | COL3A1 | genelist |
| 3 | AKR1B1 | genelist | 1 | CXCL11 | genelist |
| 3 | PLAU | genelist | 1 | CXCL2 | genelist |
| 3 | ADRA2C | genelist | 1 | DCAF5 | genelist |
| 3 | BCL2 | genelist | 1 | CHEK2 | genelist |
| 3 | BAX | genelist | 1 | CLDN4 | genelist |
| 3 | JUN | genelist | 1 | PPARA | genelist |
| 3 | CASP3 | genelist | 1 | PPARD | genelist |
| 3 | RELA | genelist | 1 | HSF1 | genelist |
| 3 | AKT1 | genelist | 1 | CRP | genelist |
| 3 | NR1I2 | genelist | 1 | CXCL10 | genelist |
| 3 | MOL004540 | mollist | 1 | CHUK | genelist |
| 2 | PDE10A | genelist | 1 | SPP1 | genelist |
| 2 | MMP2 | genelist | 1 | RUNX2 | genelist |
| 2 | MMP9 | genelist | 1 | RASSF1 | genelist |
| 2 | ADH1C | genelist | 1 | E2F1 | genelist |
| 2 | ADRA2A | genelist | 1 | E2F2 | genelist |
| 2 | MAOA | genelist | 1 | ACPP | genelist |
| 2 | CTRB1 | genelist | 1 | CTSD | genelist |
| 2 | CASP9 | genelist | 1 | IGFBP3 | genelist |
| 2 | CASP8 | genelist | 1 | IGF2 | genelist |
| 2 | PRKCA | genelist | 1 | CD40LG | genelist |
| 2 | PON1 | genelist | 1 | IRF1 | genelist |
| 2 | MOL000622 | mollist | 1 | ERBB3 | genelist |
| 2 | ESR2 | genelist | 1 | PCOLCE | genelist |
| 2 | CDK2 | genelist | 1 | NPEPPS | genelist |
| 2 | KDR | genelist | 1 | HK2 | genelist |
| 2 | DRD5 | genelist | 1 | RASA1 | genelist |
| 2 | ADRA2B | genelist | 1 | MOL005438 | mollist |
| 2 | DRD3 | genelist | 1 | IKBKB | genelist |
| 2 | VEGFA | genelist | 1 | MAPK8 | genelist |
| 2 | CDKN1A | genelist | 1 | PPP3CA | genelist |
| 2 | AHSA1 | genelist | 1 | AKR1C3 | genelist |
| 2 | TP63 | genelist | 1 | SLPI | genelist |
| 2 | SOD1 | genelist | 1 | MAPK14 | genelist |
| 2 | MMP1 | genelist | 1 | GSK3B | genelist |
| 2 | HIF1A | genelist | 1 | CCNA2 | genelist |
| 2 | STAT1 | genelist | 1 | MOL004489 | mollist |
| 2 | CDK1 | genelist | 1 | FASN | genelist |
| 2 | HMOX1 | genelist | 1 | CAT | genelist |
| 2 | CYP1A1 | genelist | 1 | PLA2G4A | genelist |
| 2 | ICAM1 | genelist | 1 | ABCC2 | genelist |
| 2 | SELE | genelist | 1 | MTOR | genelist |
| 2 | VCAM1 | genelist | 1 | PKIA | genelist |
| 2 | CYP1B1 | genelist |  |  |  |
